# Defining the risk of first intravenous immunoglobulin unresponsiveness in non-Asian patients with Kawasaki disease

**DOI:** 10.1038/s41598-020-59972-7

**Published:** 2020-02-20

**Authors:** Maryam Piram, Martha Darce Bello, Stéphanie Tellier, Sylvie Di Filippo, Franck Boralevi, Fouad Madhi, Ulrich Meinzer, Rolando Cimaz, Celine Piedvache, Isabelle Koné-Paut

**Affiliations:** 1 Université Paris-Saclay, Univ. Paris-Sud, UVSQ, CESP, Inserm, 1018 Le Kremlin Bicêtre, France; 2 AP-HP, CHU de Bicêtre, Pediatric Rheumatology, CEREMAIA, Le Kremlin Bicêtre, France; 3 CHU de de Toulouse, Paediatric Rheumatology, Nephrology and Internal medicine, Toulouse, France; 4 Hospices civils de Lyon, Cardiology, Lyon, France; 5 CHU de Bordeaux, Dermatology, Bordeaux, France; 6 CHIC, Paediatrics, Créteil, France; 7 APHP, CHU Robert Debré, Paediatrics,Paediatric Internal Medicine,Rheumatology and Infectious Diseases, RAISE, Paris, France; 8 Department of Clinical Sciences and Community Health, University of Milan, Milan, Italy; 9 APHP, CHU de Bicêtre, Clinical Research Unit, Le Kremlin Bicêtre, France

**Keywords:** Paediatric rheumatic diseases, Vasculitis syndromes

## Abstract

About 10–20% of patients with Kawasaki disease (KD) are unresponsive to intravenous immunoglobulin (IVIg) and are at increased risk of coronary artery abnormalities (CAAs). Early identification is critical to initiate aggressive therapies, but available scoring systems lack sensitivity in non-Japanese populations. We investigated the accuracy of 3 Japanese scoring systems and studied factors associated with IVIg unresponsiveness in a large multiethnic French population of children with KD to build a new scoring system. Children admitted for KD between 2011–2014 in 65 centers were enrolled. Factors associated with second line-treatment; i.e. unresponsiveness to initial IVIg treatment, were analyzed by multivariate regression analysis. The performance of our score and the Kobayashi, Egami and Sano scores were compared in our population and in ethnic subgroups. Overall, 465 children were reported by 84 physicians; 425 were classified with KD (55% European Caucasian, 12% North African/Middle Eastern, 10% African/Afro-Caribbean, 3% Asian and 11% mixed). Eighty patients (23%) needed second-line treatment. Japanese scores had poor performance in our whole population (sensitivity 14–61%). On multivariate regression analysis, predictors of secondary treatment after initial IVIG were hepatomegaly, ALT level ≥30 IU/L, lymphocyte count <2400/mm^3^ and time to treatment <5 days. The best sensitivity (77%) and specificity (60%) of this model was with 1 point per variable and cut-off ≥2 points. The sensitivity remained good in our 3 main ethnic subgroups (74–88%). We identified predictors of IVIg resistance and built a new score with good sensitivity and acceptable specificity in a non-Asian population.

## Introduction

Kawasaki disease (KD) is the leading cause of acquired heart disease in childhood in developed countries^1^. The level of coronary artery involvement mainly determines the prognosis of this systemic vasculitis affecting predominantly young children, although pericarditis, myocarditis and valvular dysfunction are not uncommon^1,2^. Occasionally, KD can be complicated during the acute phase by shock syndrome^3^, macrophage activation syndrome^4^, or myocardial infarction^1^. Although the mortality rate is relatively low during the acute phase, sudden death due to myocardial ischemia could occur many years later in children or adults with coronary artery sequelae^1^.

The efficacy of early treatment with intravenous immunoglobulin (IVIg) is well established^5,6^ and has reduced the prevalence of coronary artery abnormalities (CAAs) from 26–30% to 2.5–5% at 1 month after disease onset^6,7^. However, 30% to 40% of KD patients develop coronary dilatations within the first days of the disease^8^. In addition, approximately 10–20% of KD patients is considered resistant to IVIg and has been shown at increased risk of CAAs^1,9^. Therefore, early identification is critical to initiate more aggressive therapy^10,11^.

Several scoring systems have been developed in Japanese populations to predict resistance to IVIg therapy and show good sensitivity (77–86%) and specificity (67–86%)^12–14^. However, they lack sensitivity in North American^15,16^, European^17–19^ and other Asian populations^20,21^. Attempts to develop a scoring system with better performance in American multiethnic and Israeli populations were unsuccessful^16,22^. Moreover, reliable tools to detect children at high risk of IVIg resistance are essential because this population seems to have increased recently, from 7% to 23% in Japan^23^ and 10–20% to 38% in San Diego, USA^16^.

Data on children with KD resistant to IVIg in Europe are scarce. In addition the definition of IVIg resistance based on persistence or recrudescence of fever after completion of IVIG infusion is not homogenous with a period of observation varying from 24 h to 36 h^1,24,25^. In this study, we analyzed a large multiethnic French population of children with KD. To remain the most in keeping with the current practice, we investigated the necessity of a second-line treatment including possible IVIg re treatment after initial standard of 2 g/kg of IVIg, then we tested the accuracy of the Japanese scoring systems to predict IVIg resistance. Finally we attempted to build a new scoring system predicting secondary treatment in our large group of non-Asian patients with KD.

## Methods

### Population

Data were extracted from the Kawanet database, a national clinical and biological repository aiming to define the characteristics of KD in France. Patients admitted for KD in 65 French pediatric centers between January 1, 2011 and March 31, 2014 were prospectively or retrospectively enrolled in Kawanet by use of an online electronic case report form (e-CRF) implemented in a Web-secured system (CLEAN Web). The e-CRF contains demographic, clinical, biological, and radiologic information associated with treatments and 3-month follow-up.

The study population was limited to children (≤18 years) and was classified according to the American Heart Association (AHA) criteria in 3 groups: complete KD (fever plus at least 4 of 5 clinical criteria: exanthema, cervical adenitis, conjunctival injection, modification of oral mucosa or modification in extremities), incomplete KD (fever plus <4 clinical criteria and CAAs documented by echocardiography), and others not meeting those criteria. Records for patients in the last group were reviewed during a consensus conference with a nominal group technique by 6 experts from 5 centers (2 pediatricians, 2 pediatric rheumatologists, 1 pediatric infectious disease specialist and 1 pediatric dermatologist) to determine whether patients had probable or a doubtful KD. Participants were given complementary clinical, biological and radiological information on submitted cases. Consensus was obtained if 5/6 experts (83%) provided the same response. Some patients were unclassifiable due to missing data or lack of consensus. Patients with probable KD were taken into account in analyses but those unclassified and doubtful KD were excluded.

### Outcome measure and definitions

We analyzed demographic, clinical, biological and radiologic factors associated with unresponsiveness to IVIg. To avoid possible biases and controversies regarding the definition of IVIG resistance, we evaluated current practice decisions and analyzed in our KD cohort, the need for a second course of IVIg or second-line treatment (after the first IVIg infusion) with corticosteroids or anti-tumor necrosis factor agent.

CAAs were defined by detection of a coronary artery dilatation or aneurysms on 2-D echography at diagnosis or early follow-up. Because Z-scores were not available in most cases, coronary lesions were considered internal lumen diameter >3 mm in children <5 years old or >4 mm in children ≥5 years old, internal diameter of a segment measuring ≥1.5 times that of the adjacent segment; or a clearly irregular coronary lumen according to the Japanese Ministry of Health criteria^12,14,26^. Data on CAAs were reviewed on original echocardiographic reports requested to investigators. Other cardiac complications such as myocarditis, pericarditis, and heart failure were also reported.

Ethnicity was defined by origin of parents and classified into 5 groups: European Caucasian, North African/Middle Eastern, African/Afro-Caribbean, and Asian (Far East), or mixed (children with parents from different areas). Follow-up was defined by the number of days from diagnosis to the last visit in the center.

Kobayashi, Egami and Sano scores which predict resistance to treatment in Japanese population were studied in our population and in ethnic subgroups. The Egami score (0 to 6 points) includes age at diagnosis ≤6 months (1 point), alanine aminotransferase (ALT) level ≥80 IU/L (2 points), platelet count ≤30.10^4^ IU/L (1 point), C-reactive protein (CRP) level ≥8 mg/dl (1 point), and illness days ≤4 days (1 point), with cut-off ≥3^12^. The Sano score (0 to 3) includes CRP level ≥7 mg/dl (1 point), total bilirubine ≥0.9 mg/dl (1 point) and aspartate aminotransferase (AST) level ≥200 IU/L with cut-off ≥2^13^. The Kobayashi score (0 to 11 points) includes sodium level ≤133 mmol/L (2 points), illness days ≤4 days (2 points), aspartate aminotransferase (AST) level ≥100 IU/L (2 points), neutrophil count ≥80% (2 points), platelet count ≤30.10^4^ IU/L (1 point), CRP level ≥10 mg/dL (1 point), and age ≤12 months (1 point). We investigated the accuracy of the Kobayashi score using a cut-off ≥4 described in the original study published in 2006 in patients treated with IgIV 1 g/kg for 2 consecutive days as well as a cut-off ≥5 as described in the external validation study published in 2011 comparing children treated with IgIV 1 g/kg for 2 consecutive days and 2 g/kg in a single infusion^14,27^.

#### Statistical analyses

Descriptive statistics were used for demographic, clinical, biological and radiologic data. Continuous data are described with mean± SD and categorical data with number (%). Data were compared by chi-square test or Fisher’s exact test for categorical variables and Student’s test or Mann-Whitney test for continuous variables. Two quantitative variables (age, and time to treatment) were transformed into clinically relevant classes: age, <1 year, 1–4 years and >5 years; and time to treatment (time between the first day of fever and IVIg treatment), <5 days, 5–10 days and >10 days. Quantitative variables (laboratory variables) were transformed into binary variables by using the receiver operating characteristic (ROC) curve. To obtain the optimized cut-off value, the distance between the point (0,1) and any point on the ROC curve was minimized. The clinical relevance of the threshold was discussed. We estimated odds ratios (ORs) and 95% confidence intervals (CIs). Multivariate analysis involved logistic regression models to identify factors associated with IVIg unresponsiveness. Variables significant at p ≤ 0.20 on univariate analyses were included in the stepwise selection. Potential interactions between variables were tested. The best model was determined by using Akaike Information Criteria. We built a scoring system based on our multivariate analysis for the IVIg resistance model. For each patient, variables retained in the model determined number of criteria. ROC curves were used to obtain the optimized cut-off value for the score maximizing sensitivity and specificity to distinguish unresponsive and responsive patients to the first IVIg infusion. Sensitivity, specificity for our score and the Kobayashi, Egami and Sano scores which predict unresponsiveness to treatment in Japanese populations were studied in our population and in ethnic subgroups.

Statistical analyses involved use of SAS v9.3 (SAS Institute, Cary, NC). All tests were two-tailed, with p < 0.05 considered statistically significant.

### Ethics

The study protocol followed ethics guidelines (CPP no.CO-10–002) and was approved by the Comité Consultatif sur le Traitement de l’Information en matière de Recherche dans le domaine de la Santé (Advisory Committee on Information Processing in Research in the Field of Health, no.10.155bis) and the Commission Nationale de l’Informatique et des Libertés (National Commission of Informatics and Freedom, no. DR-2010-032). Informed consent was obtained from parents or legal guardians of children included in the study.

## Results

**Figure 1:**
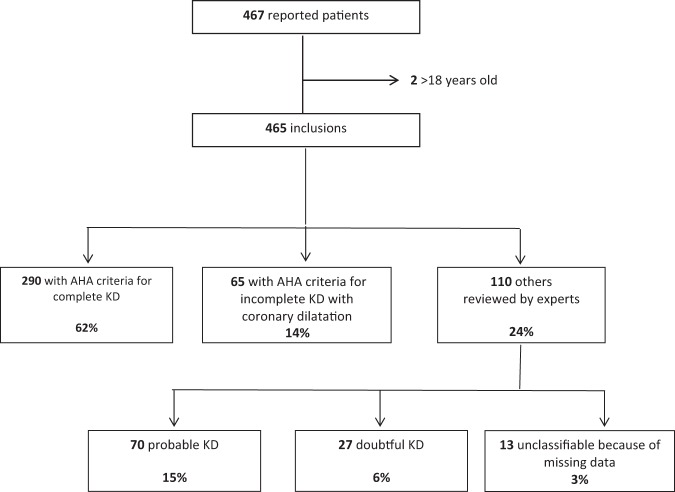
Flow of ascertainment of the 467 cases with Kawasaki disease (KD) and classification according to American Heart Association (AHA) criteria and experts.

Overall, 84 physicians reported 467 patients from 65 French centers. Two adult patients were not included in the analyses. A total of 355 patients (76%) fulfilled criteria for complete or incomplete KD. Among the 110 other patients, records for 13 were not usable because of missing data. The 97 remaining patients were classified as probable KD, 70 (72%), and doubtful KD, 27 (28%): meaning that in 27/452 (6%) cases, the diagnosis of KD was challenged by experts (Fig. 1).

### Characteristics of KD patients

For the 425 patients classified with KD (AHA + experts confirmation), 242 were boys. The mean age at disease onset was 2.8 ± 2.4 years (range 0.1 to 14.4) and mean delay to IVIg treatment 6.6 ± 3.6 days (Table 1). As expected, by definition, the frequency of clinical manifestations and coronary abnormalities differed among complete, incomplete, and probable KD. Patients with incomplete or probable KD were younger than those with complete KD (*P* = *0.006*), and although mean delay to treatment was almost the same for the 3 subgroups, patients with incomplete KD were more frequently treated after 10 days of fever (22% vs. 11% for complete KD and 7% for probable KD) (*P* = 0.02). Only 3/425 had initial combined therapy with IVIG plus steroids.

**Table 1:** Characteristics of the 425 patients with Kawasaki disease (KD) according to American Heart Association criteria and experts.

Variables	Whole KD population n = 425	Complete KD n = 290	Incomplete KD n = 65	Probable KD n = 70	p
Male/female, no (sex ratio)	242/182 (1.3)	164/125 (1.3)	38/27 (1.4)	40/30 (1.3)	0.97
Age at disease onset, years (mean ± SD)	2.8 ± 2.4	3.0 ± 2.5	2.4 ± 2.2	2.2 ± 1.6	**0.006**
Delay from fever onset to diagnosis, days (mean ± SD)	6.0 ± 3.1	5.8 ± 3.0	6.9 ± 3.5	5.9 ± 2.8	0.05
Delay from fever onset to IVIg, days (mean ± SD)	6.6 ± 3.6	6.4 ± 3.3	7.7 ± 5.2	6.5 ± 2.7	0.10
Follow-up, days (mean ± SD)	35.7 ± 55.6	33.6 ± 48.5	42.7 ± 75.1	37.7 ± 61.0	0.78
Clinical manifestations, n (%)*					
Modifications of extremities	298/424 (70%)	235 (81%)	28 (44%)	35 (50%)	** < 0.001**
Diffuse exanthema	320/399 (80%)	252 (91%)	30 (50%)	38 (62%)	** < 0.001**
Conjunctival injection	374/421 (89%)	282 (97%)	40 (63%)	52 (78%)	** < 0.001**
Cervical adenitis >1.5 cm	219/397 (55%)	186 (66%)	20 (34%)	13 (22%)	** < 0.001**
Modifications of oral mucosa	392/418 (94%)	288 (99%)	45 (74%)	59 (88%)	** < 0.001**
Cheilitis	350/413 (85%)	264 (92%)	35 (57%)	51 (78%)	** < 0.001**
Raspberry tongue	207/371 (56%)	151 (60%)	19 (32%)	37 (63%)	** < 0.001**
Erythema of the oral mucosa	275/383 (72%)	207 (79%)	30 (52%)	38 (60%)	** < 0.001**
**Maximum CRP level (mean ± SD)**	156 ± 98	157 ± 99	157 ± 111	152 ± 82	0.97
Cardiac complications, n (%)*					
Coronary dilatation	163/418 (39%)	98 (35%)	65 (100%)	0 (0%)	** < 0.001**
Myocarditis	13/402 (3%)	7 (3%)	3 (5%)	3 (5%)	0.51
Pericarditis	75/413 (18%)	50 (18%)	14 (22%)	11 (16%)	0.67
Acute heart failure	4/407 (1%)	3 (1%)	1 (2%)	0 (0%)	0.65
Treatment regimen, n (%)*					
IVIg	420/425 (99%)	287 (99%)	64 (99%)	69 (99%)	1.00
Aspirin	398/412 (97%)	270 (97%)	63 (97%)	65 (96%)	0.92
Treated ≥10 days after fever onset	50/418 (12%)	31 (11%)	14 (22%)	5 (7%)	**0.02**
Unresponsiveness to the first IVIg course	84/348 (23%)	63 (26%)	19 (31%)	12 (20%)	0.37

*Denominators excluded patients with missing or irrelevant information.

IVIg, intravenous immunoglobulin; CRP, C-reactive protein.

### Ethnicity

Among the 425 patients classified with KD, 236 (55%) were European Caucasian descendants, 50 (12%) North African/Middle Eastern, 43 (10%) African/Afro-Caribbean, 11 (3%) Asian and 47 (11%) mixed (Table 2). The ethnicity was undetermined in 38 patients (9%). As compared with European Caucasian patients, African/Afro-Caribbean patients less often had cervical adenitis (OR: 0.35, 95% CI 0.17–0.71) and patients with dark skin more often had erythema of the bottom (OR: 2.9, 95% CI 1.4–5.7 for African/Afro-Caribbean and OR: 1.9, 95% CI 1.0–3.8 for North African/Middle Eastern) and perinea desquamation (OR: 9.0, 95% CI 4.3–18.7 for African/Afro-Caribbean and OR: 2.9, 95% CI 1.3–6.3 for North African/Middle Eastern). Time to treatment, necessity of second line treatment after initial IVIG, and cardiac complications were similar in the 3 ethnic subgroups.

**Table 2:** Characteristics of patients with KD by ethnic background.

Variables	European Caucasian	North African/Middle Eastern	African/Afro-Caribbean	P
	N = 236	N = 50	N = 43	
**Demographics**				
Male/female, no. (sex ratio)	136/100 (1.4)	29/21 (1.4)	23/20 (1.2)	0.87
Age at disease onset, years (mean ± SD)	3.0 ± 2.6	2.9 ± 2.0	2.1 ± 1.6	0.16
Delay from fever onset to diagnosis, days (mean ± SD)	6.1 ± 3.1	6.1 ± 3.2	5.8 ± 2.6	0.92
Clinical manifestations, n (%)*				
Modifications of extremities	159 (69)	33 (67)	35 (81)	0.22
Diffuse exanthema	181 (82)	41 (85)	31 (72)	0.24
Conjunctival injection	211 (90)	43 (86)	38 (88)	0.67
Cervical adenitis >1.5 cm	126 (57)	28 (62)	13 (32)	**0.006**
Modifications of oral mucosa	219 (94)	46 (96)	41 (98)	0.60
Cheilitis	189 (82)	43 (90)	38 (90)	0.20
Raspberry tongue	106 (51)	27 (61)	23 (62)	0.27
Erythema	146 (69)	33 (77)	31 (74)	0.49
Erythema of the bottom	55 (27)	17 (41)	20 (51)	**0.005**
Perineal desquamation	27 (13)	13 (31)	25 (58)	** < 0.001**
**Blood tests (mean** ± **SD)**				
Maximum CRP level (mg/L)	150 ± 99	144 ± 102	193 ± 117	0.09
Albumin level (g/L)	32 ± 8	33 ± 6	27 ± 5	**0.002**
**Cardiac complications**, n (%)*				
Coronary artery anomalies	89 (38)	16 (33)	17 (40)	0.70
Myocarditis	7 (3)	2 (4)	0 (0)	0.60
Pericarditis	42 (18)	8 (17)	9 (21)	0.84
**Treatment**				
Delay from fever onset to IVIg, days (mean±SD)	6.6 ± 3.1	7.2 ± 4.5	6.7 ± 5.5	0.56
Time to treatment >10 days, n (%)	30 (13)	9 (18)	3 (7)	0.27
Unresponsiveness to IVIg, n (%)	60 (26)	9 (19)	10 (23)	0.55

*Denominators excluded patients with missing or irrelevant information.

IVIg, intravenous immunoglobulin; CRP, C-reactive protein.

### Unresponsiveness to IVIg

Information on response to the first IVIG treatment was available for 415/425 patients (98%) with KD; 92 (22%) needed second-line treatment. On univariate analysis, IVIg unresponsiveness was strongly associated with clinical or radiological hepatic or cardiac involvement (Table 3). Biologically, IVIg unresponsiveness was associated with inflammation (high CRP and procalcitonin levels, leukocytosis, low albumin level and high lymphocyte count) and hepatic involvement (high AST, ALT and gamma-glutamyl transpeptidase [GGT] levels) (Table 4). Neither disease-onset age nor delay to IVIg infusion >10 days was associated with unresponsiveness to IVIg (Table 3).

**Table 3:** Factors associated with unresponsiveness to the first IVIg infusion.

Variables	Resistance to treatment			
	Number of patients (%)		Bivariate analysis	
	Yes (n = 92)	No (n = 323)	OR	95% CI
**Demographics**				
Male	55 (23)	180 (77)	1.2	0.7–1.9
Female	37 (21)	142 (79)		
Age at diagnosis, years				
< 1	28 (28)	72 (72)		
1–5	51 (19)	211 (81)	0.6	0.4–1.1
> 5	13 (25)	38 (75)	0.9	0.4–1.9
Clinical manifestations, n (%)*				
Modifications of extremities	75 (26)	210 (74)	**2.8**	**1.5–5.1**
Diffuse exanthema	65 (21)	249 (79)	0.6	0.4–1.1
Conjunctival injection	79 (22)	278 (78)	1.0	0.5–2.1
Cervical adenitis >1.5 cm	43 (20)	169 (80)	0.8	0.5–1.3
Modifications of oral mucosa	81 (21)	303 (79)	0.6	0.3–1.6
Cheilitis	76 (22)	266 (78)	1.2	0.6–2.3
Raspberry tongue	39 (20)	161 (80)	0.8	0.5–1.3
Erythema	55 (20)	215 (80)	0.8	0.5–1.4
Erythema of the bottom	30 (28)	78 (72)	**1.8**	**1.0–3.0**
Hepatomegaly	19 (56)	15 (44)	**5.5**	**2.7–11.4**
Tachycardia	28 (30)	65 (70)	1.7	1.0–2.9
Meningeal syndrome	4 (27)	11 (73)	1.3	0.4–4.1
Hypotonia	8 (33)	16 (67)	1.8	0.7–4.3
Imaging, n (%)*				
Hydrocholecystis	11(52)	10 (48)	**4.3**	**1.7–10.5**
Cardiomegaly	8 (67)	4 (33)	**7.8**	**2.3–26.5**
Coronary artery anomalies	56 (35)	105 (65)	**3.3**	**2.0–5.3**
Myocarditis	7 (54)	6 (46)	**4.6**	**1.5–14.1**
Pericarditis	24 (32)	51(68)	**2.0**	**1.1–3.4**
**Treatment, n (%)**				
Time to treatment <5 days	32 (33)	66 (67)	**2.1**	**1.2–3.4**
Time to treatment >10 days	13 (26)	37 (74)	1.3	0.6–2.5
Aspirin with anti-inflammatory dosage	85 (22)	297 (78)	1.0	0.4–2.9

*Denominators excluded patients with missing or irrelevant information.

**Table 4:** Univariate analysis of laboratory values for unresponsiveness and response to the first IVIG infusion.

	IVIg unresponsive n = 92		IVIg response n = 323		*P*
	Number of patients		Number of patients		
CRP initial (mg/L)	88	152 ± 107	291	123 ± 83	**0.04**
CRP maximum (mg/L)	78	191 ± 117	260	146 ± 90	**0.002**
PCT initial (mg/L)	28	3.8 ± 3.0	225	1.9 ± 2.4	** < 0.001**
PCT maximum (mg/L)	24	4.4 ± 3.0	248	1.9 ± 2.2	** < 0.001**
Hemoglobin level (g/dL)	90	10.9 ± 1.5	292	11.1 ± 1.3	0.62
Leukocyte count maximum (/mm^3^)	86	20377 ± 9310	289	16281 ± 7378	** < 0.001**
Lymphocyte count (/mm^3^)	79	3206 ± 4696	267	3992 ± 4118	** < 0.001**
Platelet count (x10^3^/mm^3^)	89	368 ± 156	307	379 ± 152	0.61
AST (UI/L)	74	72 ± 101	239	54 ± 61	**0.04**
ALT (UI/L)	72	99 ± 120	239	73 ± 102	**0.005**
GGT (UI/L)	58	94 ± 93	160	65 ± 72	**0.02**
Albumin (g/L)	56	30 ± 7	214	32 ± 7	0.12
Na + (mmol/L)	81	134 ± 3	266	135 ± 3	**0.02**
Urea (mmol/L)	76	4.5 ± 4.8	249	3.8 ± 4.4	**0.003**

All data are mean ± SD.

CRP, C-reactive protein level, PCT, procalcitonin; AST, aspartate aminotransferase; ALT, alanine aminotransferase; GGT, gamma-glutamyl transpeptidase.

We could estimate the Egami, Sano and Kobayashi scores (with cut-off ≥4 and ≥5) for 328, 211 and 334 patients, respectively (Table 5). All scores had poor performance in detecting unresponsiveness to IVIg in our population with a sensitivity ranging from 14 to 61%. These scores had missed respectively 37/75 (49.3%), 38/44 (86.4%), 32/81 (39.5%) and 46/80 (57.5%) patients unresponsive to IVIg. On stratification by ethnicity, Egami and Kobayashi scores had better sensitivity for African or Afro-Caribbean patients (63 to 88%) than European Caucasians (36 to 53%) or Eastern Caucasian or North African/Middle Eastern patients (33 to 71%). Sano score had poor sensitivity in all our ethnic groups. Asian children were too few to evaluate the performance of the scores.

**Table 5:** Sensitivity and specificity of Egami, Sano, Kobayashi and Kawanet scores to predict unresponsiveness to intravenous immunoglobulin in our KD population and by ethnicity.

	Egami n = 320 patients		Sano n = 211 patients		Kobayashi cut-off ≥4 n = 334 patients		Kobayashi cut-off ≥5 n = 334 patients		Kawanet Cut-off ≥2 n = 415 patients	
	Se (%)	Sp(%)	Se(%)	Sp(%)	Se(%)	Sp(%)	Se(%)	Sp(%)	Se(%)	Sp(%)
Whole KD population	51	71	14	86	61	68	43	83	**77**	**60**
European Caucasians	51	72	18	84	53	71	36	82	**74**	**57**
Eastern Caucasian or North African/Middle Eastern patients	33	76	0	89	71	68	57	93	**80**	**65**
African or Afro-Caribbean patients	71	82	20	91	88	69	63	85	**88**	**56**

Se: sensitivity; Sp: specificity.

**Figure 2:**
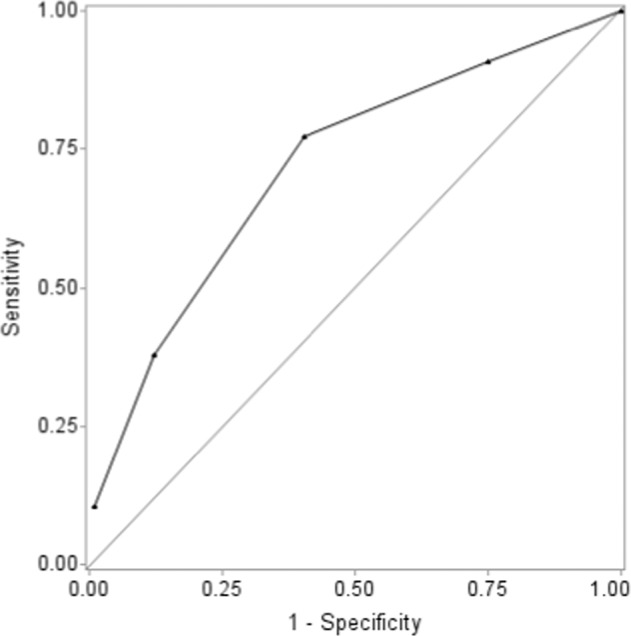
Receiver operator characteristic curve of the Kawanet score.

Because of the poor sensitivity of these Japanese scores in our population, we developed a new scoring system. On multivariate regression analysis, predictors of secondary treatment after initial IVIG were Hepatomegaly, ALT level ≥30 IU/L, lymphocyte count <2400/mm^3^ and time to treatment <5 days (Table 6). The best sensitivity (77%) and specificity (60%) of this model was with 1 point per variable and cut-off ≥2 points) with an area under the curve of 0.725. Figure 2 represents the Receiver operator characteristic curve of the Kawanet score. Sensitivity was very good with acceptable specificity in African/Afro-Caribbean population (88% and 56%) and North African/Middle Eastern population (80% and 65%) and remained acceptable in European Caucasian (74% and 57%).

**Table 6:** Multivariate logistic regression analysis of predictors of unresponsiveness to IVIg for our patients with KD.

Variables	OR (95% CI)	*P*	Points
ALT level > 30 IU/L	2.4 (1.3–4.5)	0.008	1
Hepatomegaly	3.0 (1.2–7.4)	0.020	1
Lymphocyte count < 2400/mm^3^	2.2 (1.2–4.0)	0.010	1
Time to treatment < 5 days	1.9 (1.1–3.5)	0.032	1
Total			4
Cut-off			2
Sensitivity			77%
Specificity			60%

## Discussion

Early identification of resistance to IVIg in KD is critical to initiate more effective therapies aimed to limit serious cardiac complications, especially coronary dilatations and aneurysms. Scoring systems have been established and validated in the Japanese population but they have some limitations. First, resistance to IVIG has not a universal definition in terms of persistence or length of reappraisal of fever, second, fever alone may not be the unique indicator of insufficient response as persistence of elevated CRP is associated also with cardiac complications, and finally those scores have not been validated outside the Japanese population^15–19^. We took the opportunity of the Kawanet, the widest prospective epidemiologic tool ever set up in France, a non-Asian country including multiple ethnic groups and also mixed ethnicities, to evaluate the frequency of secondary treatment after standard treatment i.e.; 2 g/kg of IVIG, and to identify the clinical, biological and radiological variables associated with the use of secondary treatments in our cohort of patients.

Kawanet aimed to analyze the degree of awareness of pediatricians and to have a thorough picture of what is called KD in France, how the disease is managed and the response to standard treatment (IVIg) but it is not a tool dedicated to calculating KD incidence (estimated 9/100,000 children <5 years of age in the Nord Pas de Calais region)^28^ or prevalence; Although the database contains exclusively e-repository data, a thorough monitoring further ensured the quality of KD cases, and all cases not satisfying the AHA criteria were reviewed and classified by consensus among KD experts, 2 independent of the Kawanet study. We registered 465 patients over a 3-year period, 76% fulfilling the AHA criteria, one third being incomplete or probable cases and significantly younger than complete KD cases (2.4 ± 2.2 and 2.2 ± 1.6 vs. 3.0 ± 2.5 years). For patients without AHA criteria, expert agreement with the referring physicians for KD diagnosis was high, 72%, reflecting a high level of knowledge of KD among French pediatricians, with a possibility of 6% over diagnosis. As a whole, the clinical characteristics of French KD patients agreed with those previously known in terms of sex ratio, age of onset, and distribution of clinical symptoms^29–31^. Patients of African/Afro-Caribbean ethnicities tended to be younger than European Caucasians and North African/Middle Eastern patients and had significantly less adenitis and more erythema and desquamation of the bottom. Unfortunately, our Asian population (n = 11) was too small to allow any comparison.

Although our KD cohort was early diagnosed and treated with an accurate regimen of IVIg, we observed a high rate of cardiac complications, especially coronary dilatations, 49%, that did not differ among our 3 ethnic populations. This discrepancy with the 26–30% coronary complications without IVIg treatment described in the literature^6,7^ is due to our definition of cardiac complications — presence of any coronary dilatation on any echocardiography ever, even if the dilatation disappeared — whereas previous rates were estimated at 1 month of disease evolution^6,7,32,33^. Because of the little published information, we could not confirm a possible reduced or increased risk of coronary dilatation in African/Afro-Caribbean patients^34,35^. Unfortunately, absence of Z-scores in Kawanet limited a more accurate analysis of cardiac complications^1^. Therefore, we focused on unresponsiveness to treatment. A relatively high proportion of our patients (22%) required secondary treatment (including IVIg re treatment) after standard treatment with 2 g/kg IVIg. Overall, the Egami, Sano and Kobayashi predicting IVIg resistance did not provide enough performance in our multi ethnic KD population. However, and to our knowledge, for the first time, we observed a better performance of two of these scores in African/Afro-Caribbean patients compared to other ethnic groups, with the exclusion of Asians. Prediction of IVIg resistance is crucial to intensify initial treatment combined with IVIg to prevent cardiac complications^12,14,36,37^, but none of the currently published scores have good sensitivity in Caucasian populations^15–19,36,38^. In our multiethnic population, we identified predictive factors of IVIg resistance based on real-life practice, and built a scoring system and obtained for the first time, good sensitivity (77%) and acceptable specificity (60%) in our non-Asian population. The sensitivity remained good in our 3 main ethnic subgroups (74 to 88%). Besides our new score, other predictors of IVIg resistance recently identified include proportion of neutrophils, hemoglobin level, CRP level, procalcitonin level, erythrocyte sedimentation rate, albumin level, creatinine level, sodium level, N-terminal pro-brain natriuretic peptide, interleukin-6 and −10 levels^19,39,40^ and baseline Z-score ≥2 or echocardiogram alteration, all found to predict high risk KD patients^19,41^.

The strengths of the Kawanet study are the wide data collection, which was predominantly prospective, the multiethnic distribution of patients, and the involvement of independent experts for case adjudication. In addition, the definition of IVIG unresponsiveness was based on current practice data rather than on any potentially controversial definition. However, the limitations include selection biases: adult patients were not included, potentially most severe patients in intensive care units were not recruited, the recruitment was voluntary and finally, our study could not compare Asian and non-Asian patients. In order to minimize risk of aneurysms associated with unresponsiveness to IVIg^19^, we maximized sensitivity of the score despite an acceptable slight decrease in specificity.

## Conclusion

We identified predictors of IVIg unresponsiveness in our cohort of KD patients and built a new score with good sensitivity and acceptable specificity in the non-Asian population. Our score, which should be tested in other multiethnic KD cohorts, is sensitive enough to be clinically useful in making decisions about initial and alternative treatments in non-Asian populations.
